# Percutaneous Closure of the Left Atrial Appendage in Atrial Fibrillation, Second Thoughts?

**DOI:** 10.4021/cr159w

**Published:** 2012-03-20

**Authors:** Philipp Wagdi, Frank Salzer

**Affiliations:** a HerzZentrum Hirslanden, Zurich, Switzerland

**Keywords:** Left atrial appendage closure, Percutaneous, Atrial fibrillation

## Abstract

Life expectancy of the population is steadily increasing world wide. Consequently, the incidence and ultimately the prevalence of atrial fibrillation (AF) and it’s sequelae will be rising proportionately. It is estimated that 3-5% of persons above 65 years of age have chronic AF, 30% of which will suffer at least one stroke. On the other hand, chronic AF is responsible for about 20% of all cerebrovascular accidents. Predictors of stroke in AF have been defined by the CHADS2 score, and in these patients, oral anticoagulation has been the cornerstone of thromboembolic disease prevention. Because elderly patients have an increased risk of bleeding complications even under the newer antagonists of Factor Xa and direct Thrombin inhibitors, percutaneous occlusion of the left atrial appendage (LAA) as the main thrombogenic source offers an attractive alternative to permanent anticoagulation. This promising new therapeutic approach is put into clinical real world perspective.

## Introduction

Succesful surgical and interventional treatment approaches for serious structural heart disease have led to a continuing rise in life expectancy in the last decades. In parallel, there has been an increase in the incidence and prevalence of chronic conditions as heart failure and atrial fibrillation (AF). More than 3% of persons above 65 years suffer from chronic AF, and roughly a third of these patients suffer at least one cerebrovascular event in their lifetime [1, 2]. In these patients, oral anticoagulants as Coumadin derivatives have constituted the mainstay of prevention of thromboembolic events [2-5], despite an incidence of potentially serious bleeding events of 3-4% per year [6]. This knowledge has provided the basis for considering an interventional alternative that might obviate the need for chronic oral anticoagulation [7].

**Figure 1:**
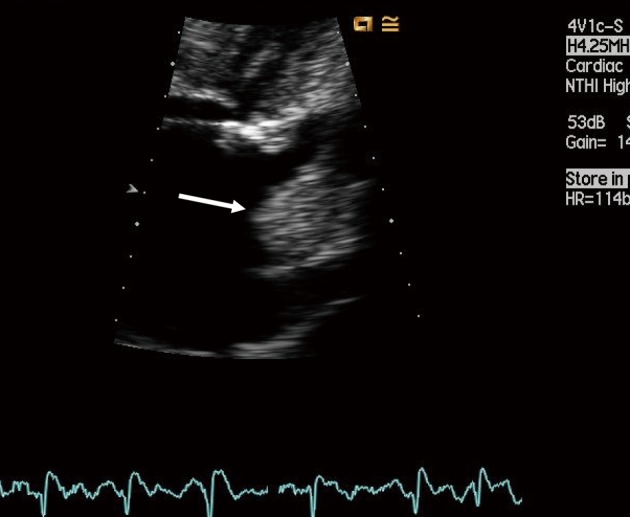
Large thrombus protruding from the left atrial appendage (arrow) as seen by transesophageal echocardiography.

In recent onset AF, the focus is on treating the underlying disease and restoring sinus rhythm by medication, cardioversion or interventional pulmonary vein isolation using catheter ablation [2, 8]. Past that stage, the therapeutic goal will shift to heart rate control either by medication or ablation of the AV node after insertion of a ventricular pace maker, as well as preventing thrombus formation in the left atrial appendage (Fig. 1) with subsequent systemic embolisation [2]. In recent years, direct Thrombin inhibitors and Factor Xa antagonists [9, 10] have been proposed as a valid alternative to conventional oral anticoagulation. Undeniably, Factor Xa and Thrombin antagonists do present some attractive properties. Because of their favourable pharmacodynamic profile including bioavailability, half life, metabolism and elimination, these drugs offer a predictable clinical effect, without the need for periodic monitoring of the international normalized ratio (INR) as was the case for Coumadin derivatives. These new drugs may thus provide more reliable anticoagulation than Coumadins, although life long compliance is again another issue. Additionally, major bleeding risk is still a concern and the need for interrupting anticoagulation in elderly patients with high CHADS2 scores in the event of a necessary operation carries a relevant risk of thromboembolism.

## Atrial fibrillation and the left atrial appendage

**Figure 2:**
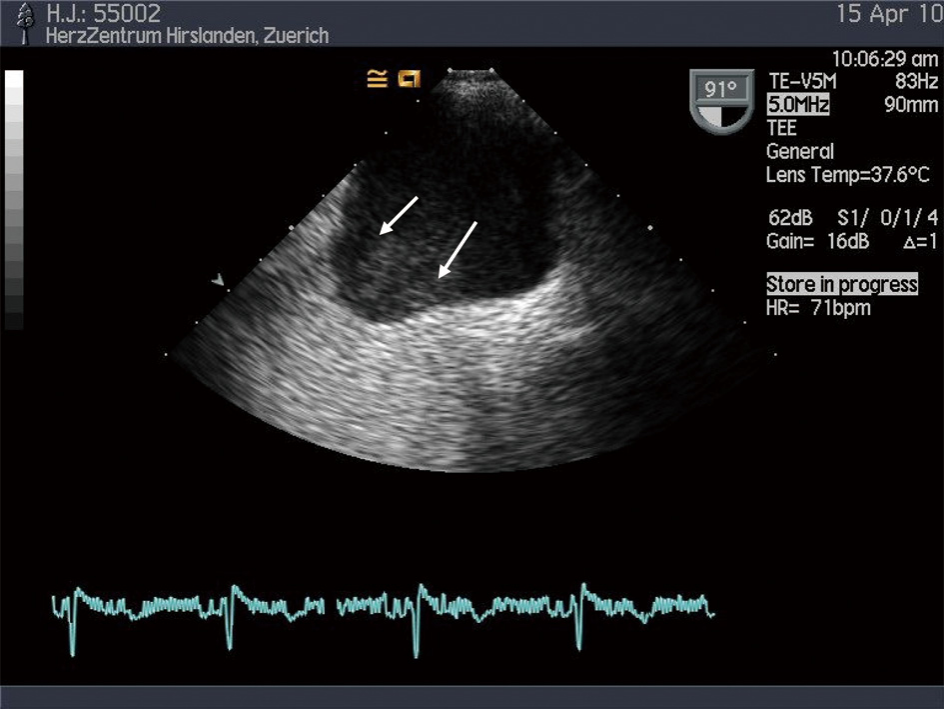
Spontaneous echo contrast (white arrows) in a dilated left atrium, hinting to high thrombogenicity.

In atrial fibrillation (AF), the left atrial appendage (LAA) has been recognized as the major thromboembolic risk, with Virchow’s triad (endothelial damage, sluggish flow and increased blood viscosity) playing an eminent role in thrombus formation [4, 11]. Figure 2 shows a case of relevant spontaneous contrast formation in the body of the left atrium proper. Surgical closure of the LAA has been advocated [11] for some time now, with results having being reviewed recently [12].

**Figure 3:**
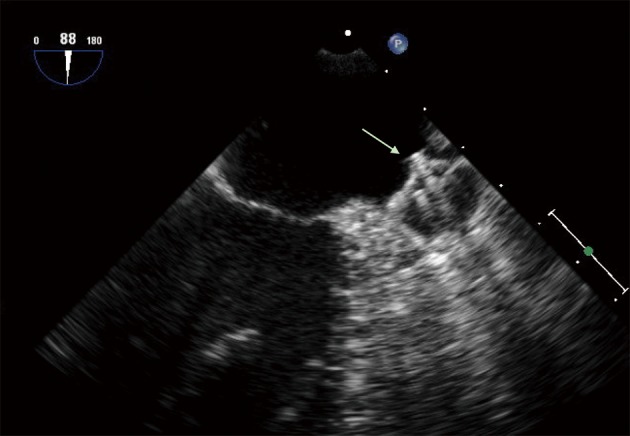
Transoesophageal echo view showing a Watchmann® device (arrow) filling the LAA lumen.

**Figure 4:**
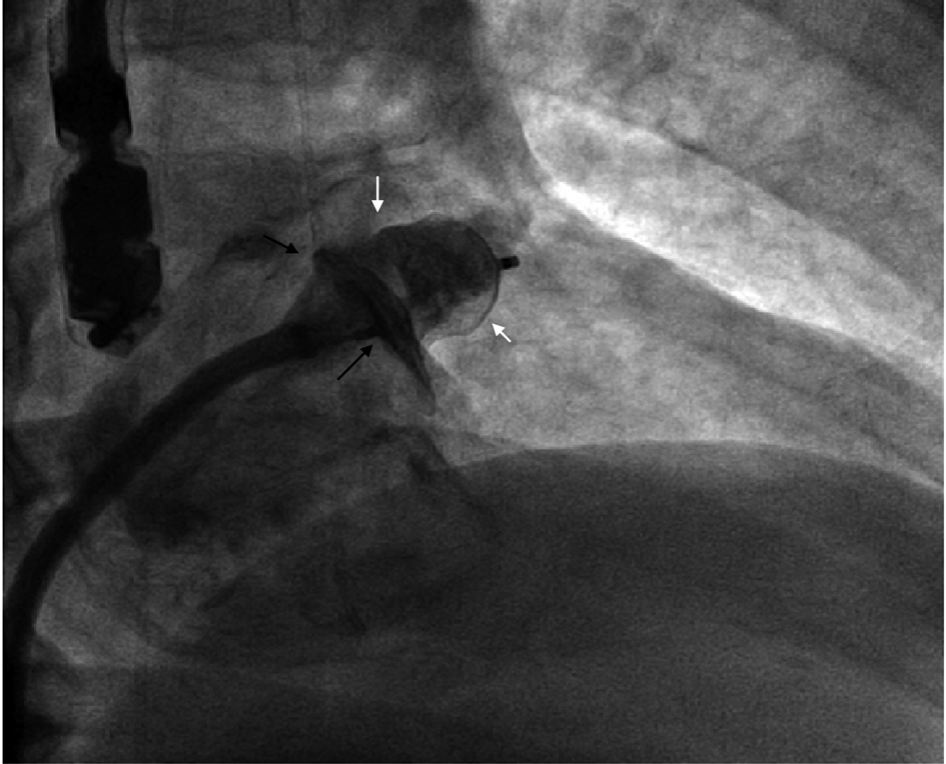
Amplatzer Cardiac Plug® LAA-occluder prior to release showing optimal lobe (white arrows) and disc (black arrows) position.

Percutaneous LAA closure has shown encouraging results, especially as experience is being gained with contemporary devices [7, 13, 14]. Two devices are currently available for LAA occlusion, both differing in background and rationale of use. The Watchmann ® (Fig. 3) is designed to be a filter, filling the LAA orifice from the inside and thus preventing thrombi from exiting. The lobe of the Amplatzer Cardiac Plug® (Fig. 4) on the other hand is not designed to fill the appendage, but to retain a disc that acts as a lid sealing the LAA from the outside. In both devices, eventual endothelialisation of the atrial surface of the occluders will prevent abluminal thrombus formation.

## Patient selection

Two of the criteria contributing to qualified patient selection are based on one hand on the refined CHADS2, the so called CHA2DS2VASc, score, assessing the individual’s statistical risk for stroke [2, 6]. On the other hand, an individual’s risk for major bleeding can be approximated by the HAS-BLED score [2, 6]. These, easily applicable, point systems help triage patients that may qualify for a LAA closure. A CHA2DS2VASc score > 2 definitely qualifies a patient for oral anticoagulation, even if he could be converted to sinus rhythm either temporarily or even on mid term. At this point, percutaneous LAA closure should not be considered as the first line of treatment yet, in a compliant patient with a score > 2, an easily maintained target INR and no history of major bleeding. A high HAS-BLED score of > 3 would on the other hand provide an argument for primary consideration for LAA closure. Increased risk of bleeding has been computed for patients with hypertension, abnormal liver or renal function, history of stroke, history of bleeding, inability to achieve stable INR values, age > 65 years, drug or alcohol consumption. The classical candidate for percutaneous LAA closure would be an elderly patient presenting a history of chronic atrial fibrillation on top of hypertensive and coronary heart disease, a cerebrovascular accident and one or more bleeding episodes under oral anticoagulation [15, 16]. In another setting, a patient should be considered for percutaneous LAA closure if a therapeutic INR would be difficult to achieve, or if they would have “quality of life” arguments against a life-time oral anticoagulation (sporting activities entailing an increased risk of trauma and bleeding).

It goes without saying that if a patient is planned to undergo cardiac surgery, surgical and not percutaneous ablation of the LAA should be done. Recently, minimally invasive videoscopic techniques have also provided excellent results [17].

## Contraindications to left atrial appendage closure and possible adverse events of the procedure

Whereas percutaneous LAA closure seems to be a safe and efficient procedure when based on sound pathophysiology, clinical indication, thoughtful intervention and sufficient experience, some consideration must be given to potential intraprocedural and follow up problems. An obvious contraindication would be the presence of an intraatrial thrombus, more specifically one in the LAA, of a cardiac tumor, an active or recent endocarditis, or an active bacterial infection (risk of device endocarditis). Obviously in patients needing life long oral anticoagulation (e.g. patients with mechanical prosthetic heart valves), a device for LAA occlusion does not make sense.

It is crucial to bear in mind that potential device-related complications are not limited to the immediate periinterventional period. Most adverse events can occur days, if not weeks or months after the procedure. Five specific complications need to be addressed, these being: 1) perforation and sometimes tamponnade in the wake of transseptal puncture (TSP); 2) tamponnade due to LAA perforation by the access sheath or by the device itself; 3) device embolization; 4) potential impingement on neighbouring cardiac structures (mitral valve annulus, aortic root, left circumflex artery, pulmonary vein orifice); 5) thrombus formation on the device.

**Figure 5:**
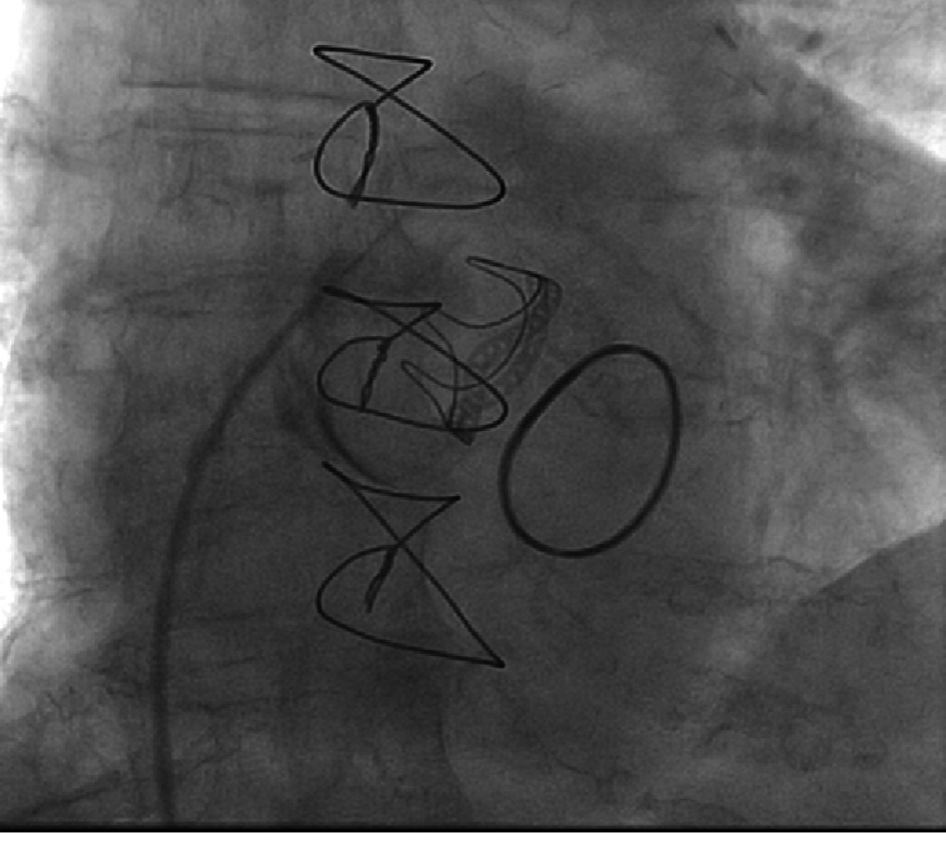
Example of an incorrect transseptal puncture. In this particular case no pericardial effusion or tamponnade results, because of extensive adhesions after cardiac surgery. Furthermore, soft injection of dye during advancement, allows early location of the ectopic position of the needle before pushing the sheath and dilator through the interatrial septum.

Laceration of a cardiac structure (aortic root, atrial wall) occurs rarely in experienced hands, especially if TSP is done under fluoroscopic and echocardiographic control. Even if inadvertent puncture occurs, a life threatening tamponnade is a rare event if the situation is recognised early, i.e. before engaging the sheath and dilator into the left atrium. In most cases of tamponnade, the procedure can be continued after pericardial drainage. In patients with prior cardiac surgery, tamponnade does not occur after inadvertent puncture (Fig. 5), because extensive adhesions prevent circumferential and hemodynamically relevant pericardial effusions.

**Figure 6:**
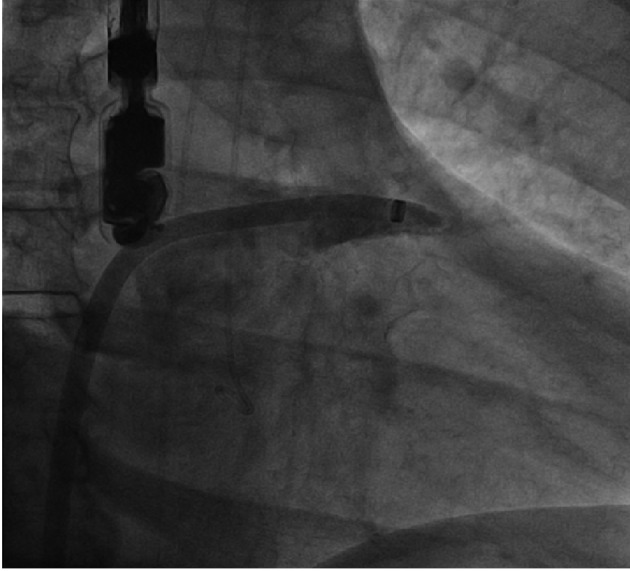
Access sheath deeply engaged in the LAA. Forceful contrast injection or inadvertent sheath movement may lead to wall laceration and tamponnade.

The LAA is a delicate, thin-walled structure. During percutaneous closure, some steps may be associated with the risk of wall injury and perforation, the first being the advancement of the access sheath into the LAA prior to contrast injection. For this reason, some operators provide the sheath with pigtail catheter at its front, to prevent trauma during advancing the sheath and contrast injection (Fig. 6). The second step is delivering the device out of the sheath. If the access sheath is not completely stable, or if it is retracted too suddenly, the device may “jump” out of the sheath and injure the LAA. Also to deep seating of the device may cause laceration of the wall by the protruding barbs.

Embolisation may occur if the chosen device size is too small, or, paradoxically too large. In the latter case, it may be squeezed out of the LAA, especially if the patient is in sinus rhythm and the LAA does contract forcefully. In most cases, the device can be percutaneously retrieved from the left atrium or the left ventricle by a snare or a biopsy grasper. If the device is lodged in the left ventricle, great care must be taken in avoiding forceful pulling, because the mitral valve chordae and papillary muscles may be avulsed. Rather, the device should be gently manipulated out of it’s trap. Rarely, a small sized device may embolize and find it’s way past the aortic valve, into the aorta, from where it will have to be delivered via an arterial access [18]. If retrieval occurs through the venous access site, it is advisable to place the snare or grasper a large sheath (12-14F) and to try capturing the device into the sheath to minimize trauma to the interatrial septum and the femoral vein.

**Figure 7:**
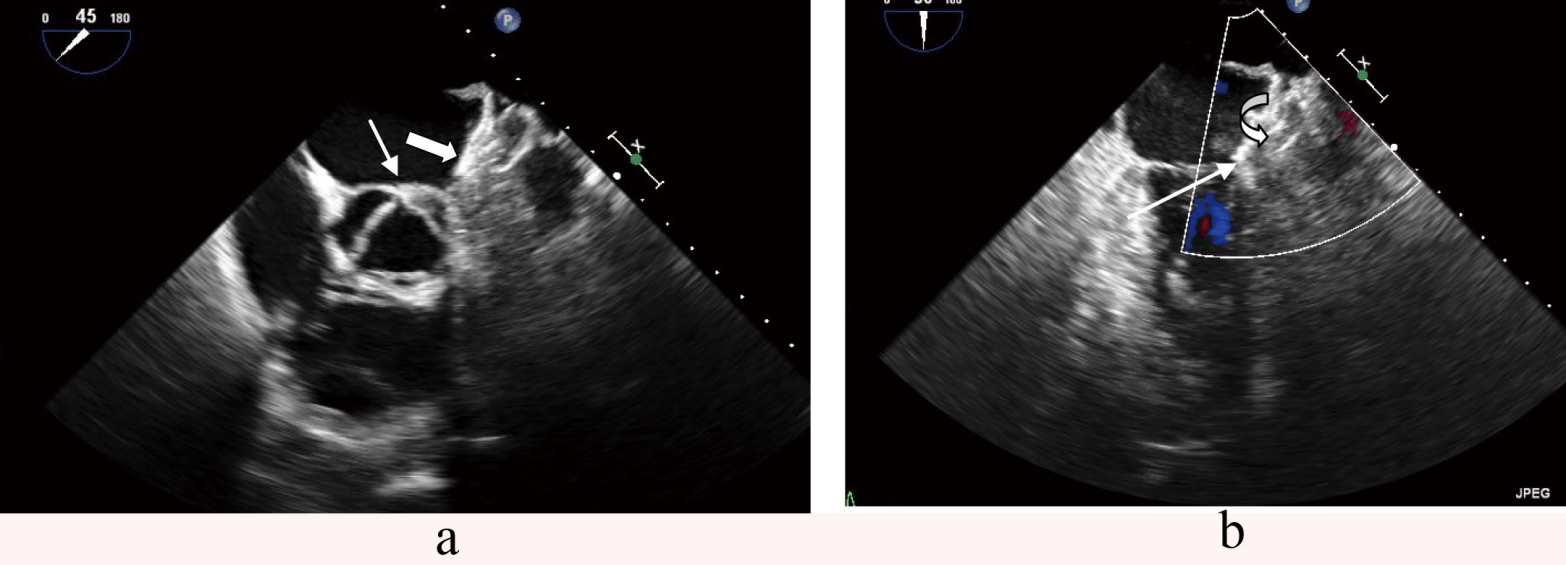
a: Adequate space between the aortic root (arrow) and the disc of the Amplatzer Cardiac Plug® (thick arrow). b: No impingement of the device (full arrow) on the mitral valve annulus (arrow).

Aortic root impingement may cause erosion (the anatomical relation is shown in Fig. 7a), while impingement on the mitral valve annulus (relation of disc to annulus is depicted in Fig. 7b) may cause erosion or interference with the valve function and regurgitation. Compression of the left circumflex artery, especially by the disc of the ACP ® device, may cause lateral ischemia. Impingement on the pulmonary artery ostium or ridge may in turn cause erosion or ostium reduction.

**Figure 8:**
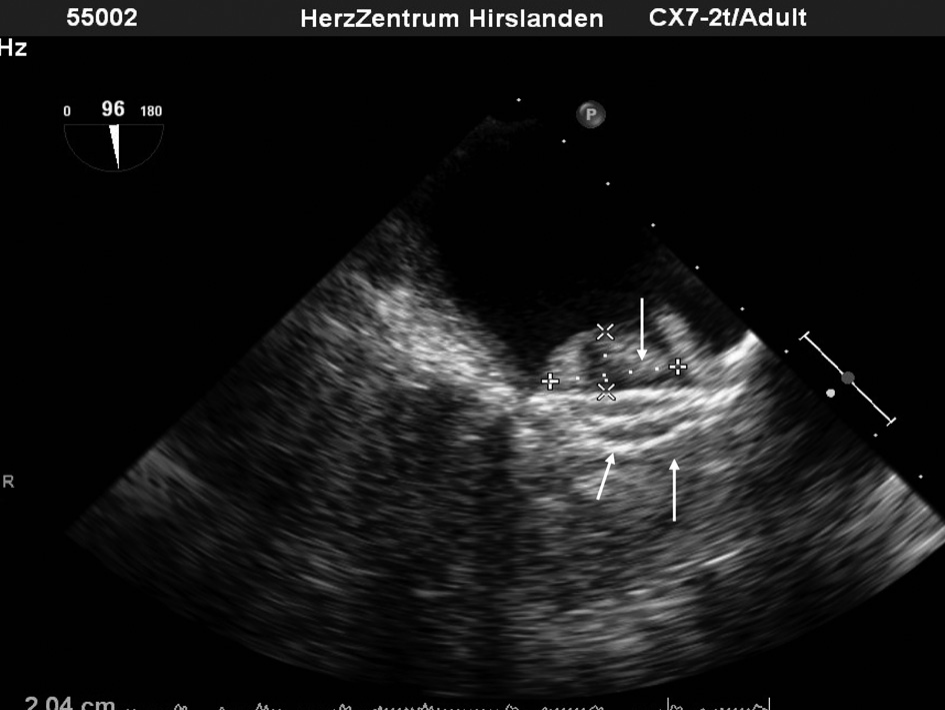
Large thrombus (x) formation on an device (arrows).

Thrombus formation on the LAA occlusion device (Fig. 8) may be underreported. Obviously, this adverse event is especially irksome, because thrombus formation is the pathology that was meant to be prevented by the intervention. A single experience - in a patient that had experienced two transient ischemic attacks followed by two incapacitating bleeding episodes under oral anticoagulation and later low molecular weight heparin only - confirmed that very large atria with extensive echo contrast formation (Fig. 1) do represent a particularly high risk. Postinterventional medication had consisted of dual platelet inhibition, we had omitted oral anticoagulation from the regimen because of the incapacitating bleeding episodes. Retrospectively, an alternative would have been to administer one of the new Factor Xa or Thrombin inhibitors for 3 months, till endothelialisation of the device surface would lower the risk of thrombus formation. In fact, an off label use of low dose Rivaroxaban® (10 mg) led to thrombus disappearance, and was well tolerated by the patient in combination with acetyl salicylic acid.

## Outlook

In summary, despite potential pitfalls and adverse events, and with increasing experience, we do not have any second thoughts concerning the validity of percutaneous LAA closure in indicated cases. We do in fact believe that the indication for the procedure could in the future be extended to include a subset of middle aged patients in whom chronic oral anticoagulation is indicated but who have not yet experienced a bleeding episode.
